# Co-Expression of DevR and DevR_N_-Aph Proteins Is Associated with Hypoxic Adaptation Defect and Virulence Attenuation of *Mycobacterium tuberculosis*

**DOI:** 10.1371/journal.pone.0009448

**Published:** 2010-02-26

**Authors:** Shyamasree De Majumdar, Deepak Sharma, Atul Vashist, Kohinoor Kaur, Neetu Kumra Taneja, Santosh Chauhan, Vijay K. Challu, V. D. Ramanathan, V. Balasangameshwara, Prahlad Kumar, Jaya Sivaswami Tyagi

**Affiliations:** 1 Department of Biotechnology, All India Institute of Medical Sciences, New Delhi, India; 2 National Tuberculosis Institute, Bangalore, India; 3 Department of Pathology, Tuberculosis Research Centre, Chennai, India; University of Hyderabad, India

## Abstract

**Background:**

The DevR response regulator is implicated in both hypoxic adaptation and virulence of *Mycobacterium tuberculosis* (*M. tb*). DevR regulon genes are powerfully induced *in vivo* implicating them in bacterial adaptation to host control strategies. A better understanding of DevR function will illumine the way for new strategies to control and treat tuberculosis.

**Methodology/Principal Findings:**

Towards this objective, we used a combination of genetic, microbiological, biochemical, cell biological tools and a guinea pig virulence assay to compare the hypoxic adaptation and virulence properties of two novel *M. tb* strains, namely, a *devR* disruption mutant, Mut1, that expresses C-terminal truncated N-terminal domain of DevR (DevR_NTD_) as a fusion protein with AphI (DevR_N_-Kan), and its complemented strain, Comp1, that expresses intact DevR along with DevR_N_-Kan. Comp1 bacteria exhibit a defect in DevR-mediated phosphosignalling, hypoxic induction of HspX and also hypoxic survival. In addition, we find that Comp1 is attenuated in virulence in guinea pigs and shows decreased infectivity of THP-1 cells. While Mut1 bacilli are also defective in hypoxic adaptation and early growth in spleen, they exhibit an overall virulence comparable to that of wild-type bacteria.

**Conclusions/Significance:**

The hypoxic defect of Comp1 is associated to a defect in DevR expression level. The demonstrated repression of DevR function by DevR_N_-Kan suggests that such a knockdown approach could be useful for evaluating the activity of DevRS and other two-component signaling pathways. Further investigation is necessary to elucidate the mechanism underlying Comp1 attenuation.

## Introduction

*Mycobacterium tuberculosis* (*M. tb*) is a versatile intracellular pathogen that has the ability to either cause active disease or produce a persistent latent infection. Tubercle bacilli exhibit dramatically contrasting phenotypes under these two conditions; during frank disease they are virulent, multiply actively and are susceptible to anti-tubercular therapy while during latent infection they display the property of non-replicative persistence, remain dormant and are quite resistant to anti-tubercular drug regimens. Therefore, an understanding of the dormant bacterial state is vital in order to devise strategies targeted towards their control and elimination. The interaction of *M. tb* with the host is likely to be dynamic and complex and to involve multiple phases of adaptation and regulatory networks. *M. tb* genome sequencing has revealed the presence of a panoply of potential regulatory molecules that comprise of transcriptional regulators, sigma factors and signaling systems including two-component systems (TCS) and eukaryotic-like serine threonine protein kinases/phosphatases [1]. All of these are likely to play a dynamic role in bacterial adaptation to the changing environmental conditions within the host.

Bacterial TCS are involved in the control of a wide variety of physiological processes ranging from nutrient uptake to virulence. TCS of *M. tb* have been intensely studied by many laboratories and as expected, several of these systems are responsible for bacterial adaptation within the host [2], [3]. One of the best characterized TCS of *M. tb* is *devRS* (also called *dosRS*). *devR* (*Rv3133c* or *dosR*) was identified as a differentially expressed gene in virulent *M. tb* H37Rv [4], [5] and it encodes DevR which is activated by transfer of phosphosignal from DevS and/or Rv2027c/DosT [6]–[8]. It is directly involved in the hypoxia-induced dormancy response [9]–[11] and also in virulence [12]–[15]. Moreover, DevR and its target genes are highly expressed in animals and cell infection models which suggests that bacteria rely on them for adaptation *in vivo* [16]–[20].

DevR is a classical response regulator which contains a N-terminal phosphorylation domain and a C-terminal DNA binding domain [5]. Phosphorylation of DevR is essential for the activation of its DNA binding function, its autoinduction and the induction of DevR regulon genes expression [21]–[23]. A novel *devR* mutant strain, Mut1, was generated serendipitously in our laboratory by an in-frame insertion of a promoterless kanamycin resistance cassette into the *devR* gene at an unique PpuMI site which results in the expression of C-terminal truncated DevR as a DevR_NTD_-AphI fusion protein (DevR_N_-Kan). The fusion protein confers kanamycin resistance to the mutant bacterium and enabled its original selection [13]. Its complemented strain, Comp1, expresses intact DevR from its native 327 bp upstream region along with DevR_N_-Kan fusion protein [13]. In the present study, we studied the properties of guinea pig-passaged Mut1 and Comp1 bacteria alongside wild-type H37Rv (WT) bacteria. We find that Mut1 bacilli exhibit a defect in hypoxic adaptation and early growth within spleen but exhibited overall virulence nearly comparable to WT bacilli. Interestingly, in Comp1 bacteria, DevR_N_-Kan competes for the activating phosphosignal resulting in a defective hypoxia adaptive response. We also find that Comp1 is attenuated in virulence. The potential implications and possible application of these findings are discussed.

## Results

### DevR_N_-Kan Inhibits HspX Induction in Comp1 Bacteria

**Figure 1:**
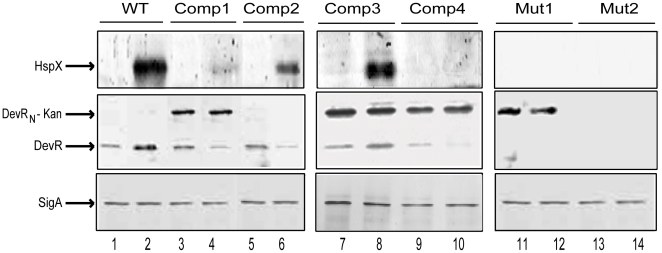
Effect of DevR_N_-Kan and full-length DevR co-expression on DevR regulon gene expression. *M. tb* lysates were electrophoresed and subjected to immunoblot analysis using polyclonal antibodies to HspX (top panel), DevR (middle panel) and SigA (bottom panel). Lanes 1, 3, 5, 7, 9, 11 and 13 represent aerobic culture and lanes 2, 4, 6, 8, 10, 12 and 14 represent 5 days standing hypoxic cultures. Anti-HspX immunoblots were developed for longer periods to visualize HspX in Comp1 bacterial lysates. Representative blots from 2 to 4 independent cultures are shown.

*hspX* is a DevR-regulated gene and its expression is a reliable marker of DevR regulon expression. HspX expression was strongly induced in hypoxic WT cultures (Fig. 1, lanes 1–2) in contrast to the lack of expression in Mut1 bacteria. Surprisingly however, HspX was only weakly expressed in Comp1 bacteria (that expresses both DevR_N_-Kan and full-length DevR proteins) under similar conditions. To correlate with this defect, DevR expression was assessed; while it was induced in hypoxic WT cultures (Fig. 1, lanes 1–2), its level declined in Comp1 bacteria (Fig. 1, lanes 3–4). Furthermore, DevR level was consistently lower relative to DevR_N_-Kan (∼55% and ∼20% under aerobic and hypoxic conditions respectively, a representative blot is shown in Fig. 1, lanes 3–4).

### The Expression Defect in Comp1 Is Ascribed to Inhibition of Signaling by DevR_N_-Kan

**Figure 2:**
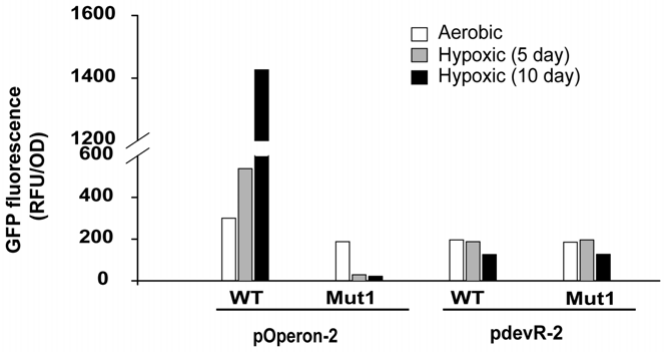
Comparison of promoter activity using GFP reporter assay. GFP fluorescence in *M. tb* WT and Mut1 cultures carrying operon (pOperon-2) and *devR* (pdevR-2) promoter constructs under aerobic and hypoxic conditions.

The skewed protein ratios (possibly due to differences in promoter strength) suggest that DevR_N_-Kan may interfere with intact DevR function in Comp1 bacteria. This hypothesis was tested by assessing HspX expression in Comp2 strain that was generated by introducing pDSDevR into a complete *devR* deletion mutant strain (Tables 1 and 2). HspX induction was restored in Comp2 (Fig. 1, lanes 5–6), indicating that the hypoxic expression defect in Comp1 was due to DevR_N_-Kan-mediated inhibition. Towards understanding the underlying basis of this defect, the promoters expressing intact DevR and DevR_N_-Kan proteins were compared since in Comp1 bacteria, full-length DevR is expressed from the complementing plasmid through its upstream promoter (as in pdevR-2) while DevR_N_-Kan is expressed from its natural genomic location (as in pOperon-2). From the GFP reporter activity it is evident that pOperon-2 displays both aerobic and DevR-dependent inducible expression under hypoxia while pdevR-2 shows constitutive and moderate activity that is independent of DevR (Fig. 2). Considering the results of immunoblotting and reporter assays, the observed decline in DevR protein level during hypoxia in Comp1 bacteria is likely to be a consequence of a defect in expression (since DevR ectopic expression from a constitutive promoter is not sensitive to induction during hypoxia). By contrast, DevR_N_-Kan levels maybe stabilized as a fusion protein and/or due to DevR expression from the inducible promoter (since Comp1 bacteria synthesize DevR, albeit at low levels).

**Table 1:** Plasmids used in this study.

Plasmid	Description	Reference
pDSdevR	*devR* gene cloned in pFPV Hyg (low copy number plasmid), DevR expressed from 327 bp *devR* upstream promoter, Hyg^r^	[13]
pJFR19	3 kb amidase promoter cloned in integrative plasmid pMV306H, Hyg^r^	[32]
pOperon-2	pFPV27 (promoter less GFP) containing operon promoter (−1454 to +12) with reference to the *devR* translational start site, Hyg^r^	This study
pdevR-2	pFPV27 (promoter less GFP) containing *devR* promoter (−390 to +164) with reference to the *devR* translational start site, Hyg^r^	[21]
pAVdevR_N_ -Kan	DNA coding for DevR_N_ - Kan fusion protein cloned in pJFR19, protein expressed from native operon promoter (described in [21]), Hyg^r^, Kan^r^	This study
pDSS578	pPROEx-HTb carrying wild type *devS* gene	D. K. Saini, Ph.D. thesis, AIIMS
pET-28-a	Vector for overexpression of His_6_-tagged recombinant proteins, Kan^r^	Novagen
pKKNKan	pET-28-a based plasmid for overexpression of DevR_N_-Kan fusion protein, Kan^r^	This study
pAVDevR	pET-28-a based plasmid for overexpression of full-length DevR protein, Kan^r^	This study

**Table 2:** *M. tb* strains used in this study.

*M. tb* strain	Description	Expression		Reference
		DevR Aer Hyp	DevR_N-_Kan Aer Hyp	
H37Rv	WT	+ +++	−−	[13]
Mut1	*devR*Δ*CTD*, expresses DevR_N_-Kan protein from its native location (fusion gene created by in-frame insertion of promoterless kanamycin resistance cassette at the PpuMI site within *devR* gene), includes entire N-terminal signaling domain of DevR [residues 1–145], Kan^r^	−−	+ ++ +++	[13], this study
Mut2	Complete Δ*devR* deletion	− −	− −	[12]
Comp1	*M. tb* Mut1 complemented with plasmid pDSDevR, Kan^r^, Hyg^r^	+ ↓	+++ +++	[13]
Comp2	*M. tb* Mut2 complemented with pDSDevR, Kan^r^, Hyg^r^	+ ↓	−−	This study
Comp3	H37Rv containing pAVDevR_N_-Kan, Kan^r^, Hyg^r^	+ ++	+ + + +++	This study
Comp4	*M. tb* Comp2 containing pAVDevR_N_-Kan, Kan^r^, Hyg^r^	+ ↓	+++ +++	This study

Aer, aerobic; Hyp, hypoxic.

+, ++ etc., relative levels of DevR and DevR_N-_Kan proteins (semi- quantitative).

−, absence of DevR.

↓, decline in hypoxic level.

The inhibitor function of DevR_N_-Kan was confirmed in two additional *M. tb* strains (Table 2). In Comp3 bacteria (generated in H37Rv background and expressing DevR_N_-Kan and WT DevR proteins, each from the native inducible promoter), HspX expression was induced (Fig. 1, lanes 7–8), indicating that DevR_N_-Kan inhibitory activity is overcome in the presence of WT DevR levels. However, HspX induction was not rescued in Comp4 bacteria (generated in a complete *devR* deletion strain that produced a skewed ratio of DevR_N_-Kan and full-length DevR proteins), akin to Comp1 bacteria (Fig. 1, lanes 9–10). Note that although DevR_N_-Kan was expressed at an elevated level from its ectopic location in Comp3 and Comp4 strains vs. from its native location in Comp1 (Fig. 1), HspX expression was consistently restored in Comp3 bacteria but not in Comp4 bacteria. Likewise, absence of HspX induction in Comp4 but not Comp2 bacteria (both in complete *devR* deletion background) is attributed to the presence of DevR_N_-Kan in the former (Fig. 1). These results establish that the hypoxic defect of Comp1 in terms of HspX expression is associated to a defect in DevR expression level.

**Figure 3:**
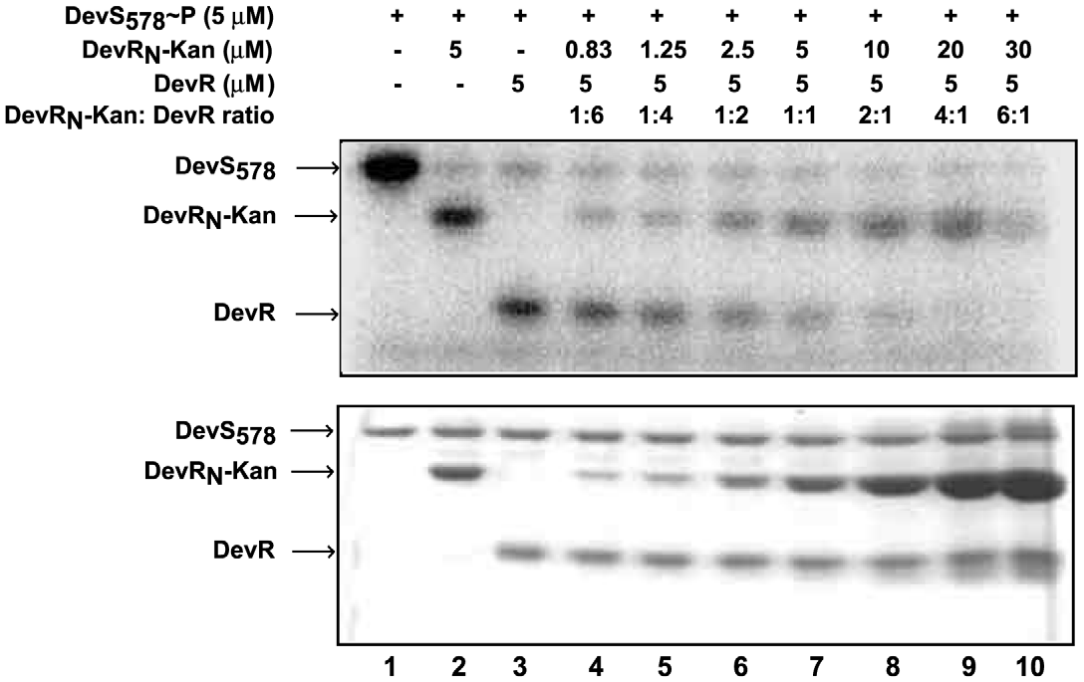
DevR_N_-Kan competes efficiently with full-length DevR for phosphosignal from DevS. Reaction mixtures containing purified DevS∼P (5 µM) plus DevR_N_-Kan (0.83 to 30 µM) and full-length DevR (5 µM) proteins were incubated at 25°C for 2 minutes. Samples were analyzed by 15% SDS-PAGE and subjected to phosphorimaging (top panel) and Coomassie blue staining (bottom panel).

We next asked whether DevR_N_-Kan competed with DevR for the activating phosphosignal in Comp1 bacteria by reconstituting the phosphosignaling reaction *in vitro*. Briefly, DevR_N_-Kan coding sequences (exactly as expressed in Mut1 bacilli) were cloned, the overexpressed protein was purified and used with full-length DevR in a DevS∼P-driven competition assay (Fig. 3). The phosphosignal was transferred to DevR and DevR_N_-Kan inhibitor with approximately similar efficiency when they were present at equimolar concentrations. Importantly, the signal was diverted majorly to the inhibitor at higher concentrations of DevR_N_-Kan, which mimics the protein ratios *in vivo*, indicating that preferential phosphorylation of DevR_N_-Kan is likely to occur *in vivo*. Moreover, in a phosphosignaling competition assay performed with DevR_N_ protein (without the kanamycin resistance cassette), similar results were obtained (not shown), thereby attributing the inhibition to DevR_N_ in the fusion protein.

All these findings, namely, (a) efficient diversion of phosphosignal to DevR_N_-Kan *in vitro*, (b) skewed DevR_N_-Kan: DevR protein ratio *in vivo* resulting in diversion of the phosphosignal to the former and, (c) defective HspX induction in Comp1 and Comp4 bacteria, conclusively establish that DevR_N_-Kan inhibits DevR signaling.

### Comp1 Bacteria Are Defective in Hypoxic Survival

**Figure 4:**
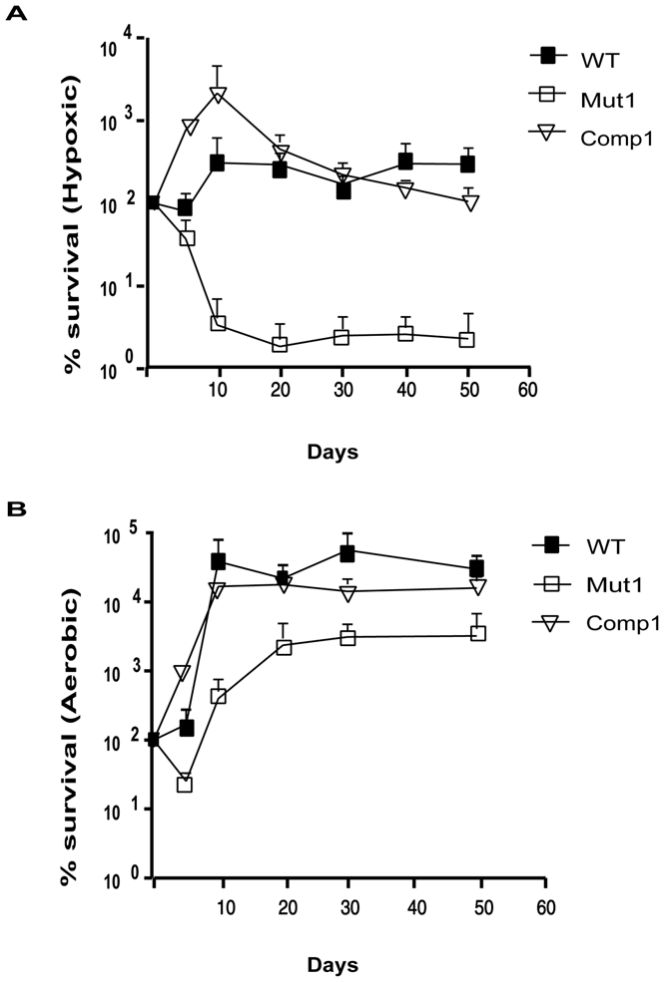
Survival of *M. tuberculosis* strains cultured *in vitro*. WT, Mut1 and Comp1 strains were grown under hypoxic (**A**) and aerobic (**B**) conditions for upto 50 days in Dubos Tween Albumin medium as described. The mean CFU ± SD determined from three independent cultures is shown as % survival with respect to number of bacteria on day zero. ▪, WT; □, Mut1 and ▿, Comp1.

As DevR plays a crucial role in the mycobacterial adaptive response to hypoxia, we evaluated the survival properties of Comp1 bacteria under hypoxia (Fig. 4). Hypoxic viability was sustained in WT bacilli and on day 50, ∼105% of the bacteria remained viable (relative to maximum CFU on day 10). By contrast, the hypoxic survival defect in Mut1 bacilli was evident from day 5 (the earliest time point when bacteria were sampled) and only ∼2% of the initial bacterial load (maximum CFU) were viable on day 50. If we compare initial and final number of bacteria, there is little difference in hypoxic viability between Comp1 and WT strains. However, under hypoxic conditions, Comp1 grew more rapidly than WT bacteria during the first 10 days and thereafter its viability was not sustained and on day 50, approximately 5% of the bacteria were viable relative to maximum CFU observed on day 10. All the strains grew at similar rates and exhibited similar viability under aerobic conditions. The hypoxia survival defect of Comp1 bacteria is attributed to an insufficient level of activated DevR. Another possible underlying reason for the defect in Comp1 bacteria is that expression of DevR from the natural genomic location and from complementing plasmids is very different and these differences may affect other proteins involved in the two-component system signaling in an unknown manner.

### *M. tuberculosis* Comp1 Strain Is Attenuated in Guinea Pigs

**Figure 5:**
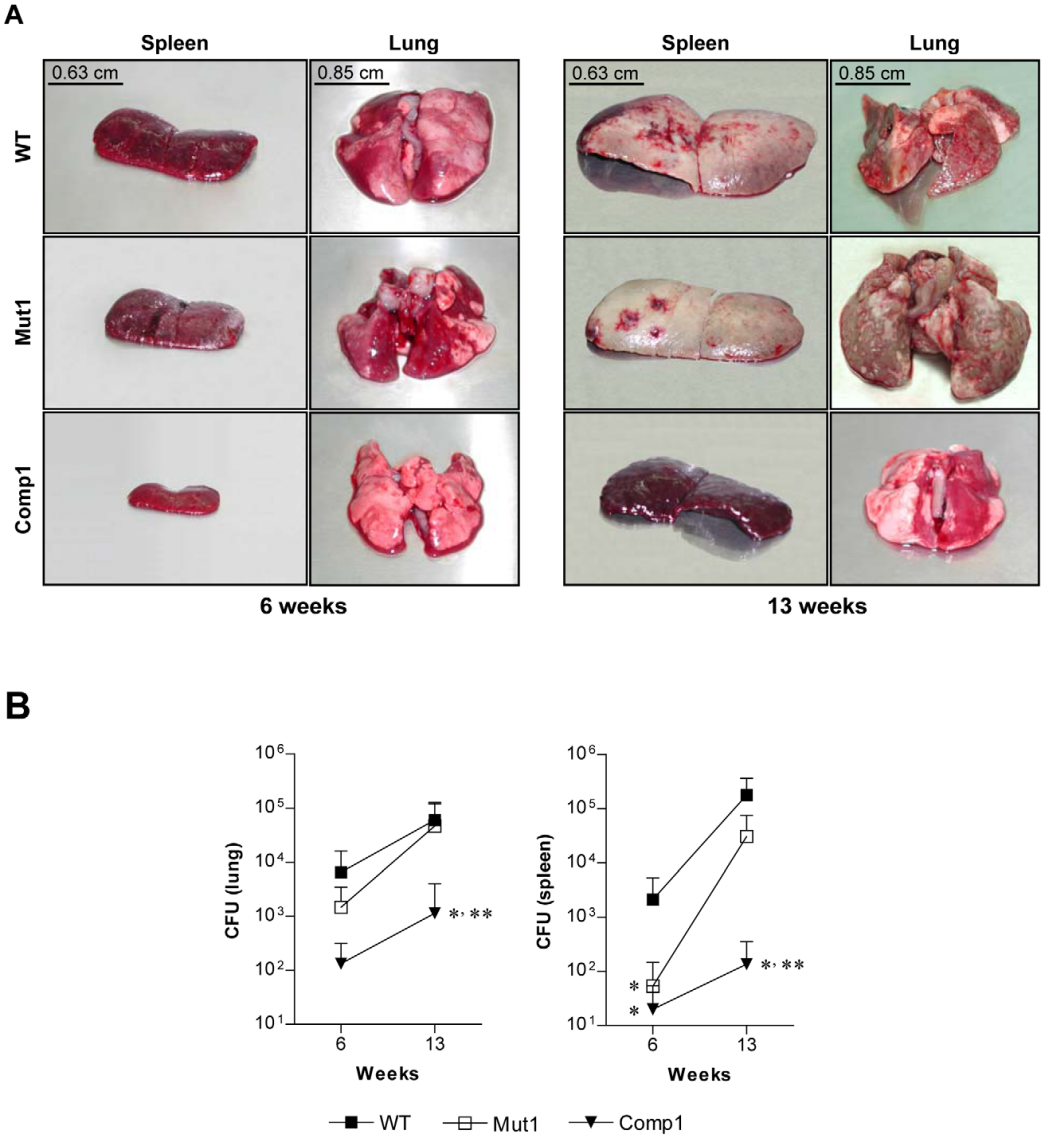
Virulence of passaged *M. tuberculosis* strains in guinea pigs. (**A**) Pictorial representation of lungs and spleens. (**B**) CFU in lungs and spleens are expressed as mean ± SD. *, ** represent P<0.05 in comparison to WT and Mut1, respectively.

Passaged Mut1, Comp1 and WT bacteria were tested in the guinea pig virulence model [24], [25]. At 6 weeks, a nearly similar number of lesions were visually scored for both the WT and mutant strains. By contrast, fewer lesions were visually scored in the Comp1 group (P<0.05 in comparison to WT and Mut1 groups, Table 3). The spleens of WT and mutant-infected groups were also significantly enlarged in comparison to Comp1 group of animals (Table 3 and Fig. 5A). The extent of lung and liver granuloma (P<0.05 in comparison to WT group) and lung CFU were lower in the Comp1 group (Fig. 5B and Table 3) and spleen CFU was lower in both mutant and complemented groups (P<0.05 in comparison to WT, Fig. 5B).

**Table 3:** Virulence comparison of passaged *M. tb* strains.

	6 weeks			13 weeks		
	WT	Mut1	Comp1	WT	Mut1	Comp1
**Visual scores** #	32.25±4.34	25.25±5.85	8±0* ^,^ **	55.2±8.25	48.66±4.96	18.5±8.73* ^,^ **
**Lung granuloma (%)**	58.75±2.39	43±15.77	25±2.04*	67.5±12.99	74.16±9.95	33.33±2.76* ^,^ **
**Liver granuloma (%)**	46.25±9.43	21.66±3.75	12.5±4.78*	68.75±5.15	80.83±3.27	28.5±7.30* ^,^ **
**Spleen weight ratios** ∧	1.08±0.25	0.87±0.40	0.26±0.05* ^,^ **	3.75±0.90	3.30±1.54	0.57±0.62* ^,^ **
**Lung weight ratios**	0.80±0.14	0.79±0.05	0.73±0.14	1.84±1.25	1.96±0.42	0.73±0.10* ^,^ **
**Liver weight ratios**	5.85±0.37	4.51±0.66	5.48±0.92	7.54±1.76	7.16±2.19	4.18±0.84* ^,^ **

# Mean total of lesion scores assigned to spleen, liver, lung and the site of injection along with its draining lymph nodes immediately after death as described [24].

*P<0.05 in comparison to WT.

**P<0.05 in comparison to Mut1.

∧ Weight ratio  =  (organ weight/body weight) ×100.

To evaluate disease progression, a second infection of 13 weeks was performed. An increase in visually scored tubercles was noted in all the groups; however, once again the number of visually scored lesions was lower in the Comp1 group (P<0.05, Table 3). Progressive splenic enlargement was noted at 13 weeks in WT and Mut1 groups but not in the Comp1 group (Table 3). CFUs in lungs and spleens also increased at 13 weeks for all the strains. Although fewer bacteria were recovered from lungs and spleen of Mut1-infected animals at both 6 and 13 weeks of infection, with the exception of a significant reduction in spleen CFU at 6 weeks (Fig. 5B), the differences were not significant compared to the WT group (Fig. 5B). However, a significant growth defect of Comp1 bacilli persisted at 13 weeks in both organs (Fig. 5B). The extent of organ granuloma correlated quite well with bacterial CFU and visual scores (Table 3). In qualitative terms, granulomas in liver were composed essentially of lymphocytes, macrophages and large numbers of epithelioid cells. In the lung, epithelioid cells were rare and the granuloma consisted mainly of lymphocytes and macrophages (not shown). There was very little necrosis in both the organs. Overall, the results of the two experiments are consistent with an attenuation of Comp1 bacteria (P<0.05 compared to WT) and a modest lowering of virulence for Mut1 bacteria that was not significant.

### Reduced Infectivity of Comp1 Bacilli in THP-1 Infection Model

**Figure 6:**
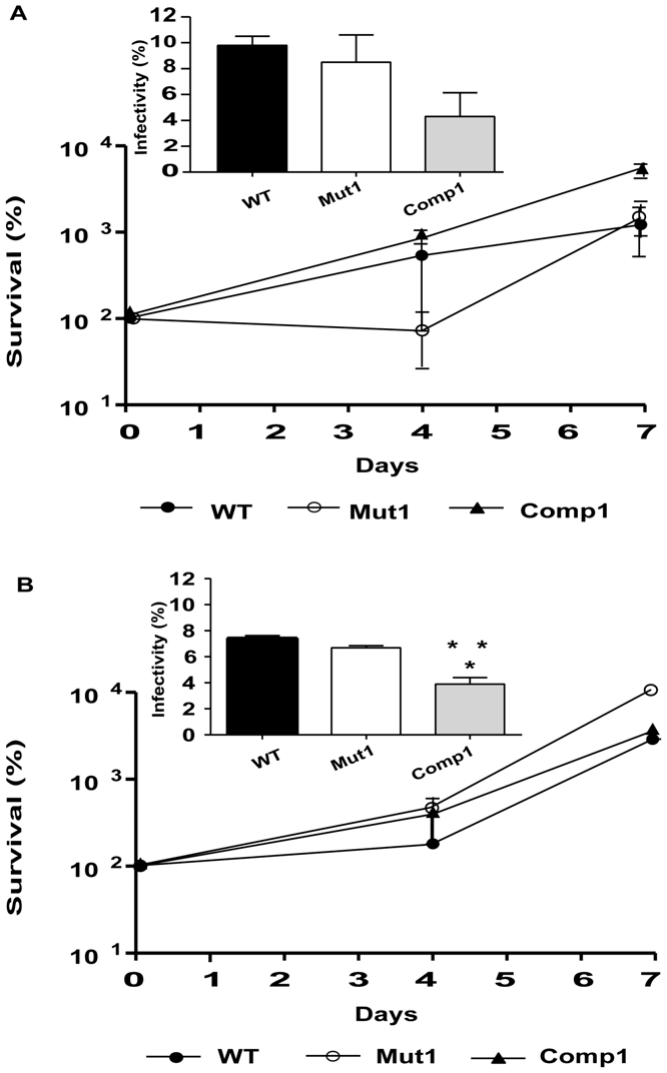
Intracellular survival of *M. tuberculosis* strains. THP-1 cells were infected with various strains (**A,** passaged and **B,** laboratory cultured strains) at a m.o.i of 1∶50 (bacterium: macrophage) and the number of intracellular viable bacteria was determined over 7 days. Results are given as the mean ± SD of 3 independent experiments. (*Inset*) Infectivity of the three strains in THP-1 cells as determined by 2 separate experiments. *, ** P<0.05 indicate significant differences in infectivity between Comp1 vs. WT and Mut1, respectively. •, WT; ○, Mut1 and ▴, Comp1.

The strains were next assessed in the THP-1 cell infection model to determine whether Comp1 bacteria possessed an intracellular survival defect (Fig. 6). Passaged and laboratory cultured organisms of all the strains exhibited quite similar intracellular survival and growth properties over a 7-day period (Fig. 6). However, Comp1 bacteria displayed a reduced ability to infect THP-1 cells in comparison to the WT and Mut1 strains (Fig. 6 insets). For the passaged strains, the infectivity rate of the WT and Mut1 strains was 9.8% and 8.5% compared to 4.3% for Comp1 bacteria. Similar observations were made with the laboratory cultured strains; only 4.3% infection was observed with Comp1 bacteria compared to 7.4% and 6.7% for WT and Mut1 strains, respectively. The macrophage infection assay performed at a higher m.o.i (1 bacterium per 10 macrophages) further confirmed the decreased infectivity of Comp1 organisms (not shown).

### Animal Passaged Mut1 Bacilli Multiply Preferentially in Lungs

In a guinea pig virulence assay performed previously, Mut1 bacilli were observed to be attenuated in terms of visually observable lesions and spleen CFUs [13]. However, Mut1 bacterial attenuation was not observed in the present study. The difference between the two studies is that the previous study was performed with Mut1 bacteria that had been repeatedly cultured *in vitro* during the generation of the mutant strain, whereas the present one was carried out with guinea pig-passaged bacteria. Since repeated *in vitro* culture of pathogenic bacteria can result in their attenuation [26], we compared animal passaged and laboratory cultured bacteria in a 6 weeks side-by-side guinea pig virulence assay to determine whether repeated laboratory culture was the underlying reason for the observed attenuation of Mut1 bacteria. Interestingly, spleen CFUs in Mut1 bacilli group were significantly lower relative to WT CFUs in both passaged and laboratory cultured bacilli (Fig. S1) and these results were consistent with our previous observations [13]. Observable lesions, liver granulomas and spleen weight ratios were also significantly lower in laboratory cultured Mut1 bacilli infected group (Table S1). This was also consistent with previous observations [13] indicating that overall pathology was decreased by laboratory passaging (P<0.05, Table S1). By contrast, passaged, and not laboratory cultured, Mut1 bacteria exhibited prolific multiplication in lung accompanied by a decrease in splenic CFU load suggesting that laboratory cultured bacilli exhibited a lung-specific defect (P<0.05, Fig. S1).

## Discussion

In this study a *devR* disruption mutant strain, Mut1, and its complemented strain, Comp1, were assessed for their hypoxia adaptability and virulence properties. The Comp1 strain is novel in that it is defective in the hypoxic response. This defect is explained by skewed expression of DevR_N_-Kan vs. intact DevR protein and an associated skewing of phosphosignaling, which likely results in insufficient availability of activated DevR.

A key finding of this study is that Comp1 bacteria are attenuated. We exclude the possibility of attenuation due to an intrinsic growth defect since Comp1 bacteria multiply normally in broth cultures and within infected THP-1 cells. However, lower infectivity of THP-1 cells suggests a scenario wherein Comp1 bacteria could be gradually cleared over multiple cycles of infection and result in significantly lower bacterial loads. Since bacteria disseminate from the site of injection in the thigh to various organs in this virulence assay [24], an infectivity and/dissemination defect could also contribute to its attenuation. However, differences in infecting dose as a possible reason for differences in virulence are ruled out since an approximately equal number of viable organisms of each strain were injected subcutaneously per animal.

Passaged Mut1-infected guinea pigs contained significantly lower spleen bacterial loads at 6 weeks and this was consistent with our previous observations [13]. Although lung CFUs were also lower in Mut1 vs. WT-infected animals, the difference was not significant. Therefore we conclude that passaged Mut1 bacilli are overall nearly as virulent as WT organisms. In contrast, the Comp1 strain was attenuated by all parameters (organ inflammation, histology, visually observable lesions and organ CFUs). Studies with various *devR* or *dosR* mutant and complemented strains have reported virulence phenotypes ranging from attenuation to hypervirulence and these variations have been attributed to differences in strain construction and the use of different models [12]–[15]. Our experiments indicate that animal passaging restores the ability of Mut1 bacilli to multiply in guinea pig lungs suggesting that a decreased capacity of laboratory cultured organisms to establish a productive lung infection is a key aspect of attenuation that could have occurred by repeated *in vitro* culturing during Mut1 construction. It was recently suggested that the variable results of various animal studies could be explained by differences in both host and infecting dose [15]. Our study provides evidence that the mode of bacterial propagation also significantly influences the virulence phenotype.

Except for the present study performed with a disruption mutant, all other investigations were performed with deletion mutant strains. The strains also vary in the expression of the co-transcribed *devS* gene; Mut1 expresses DevS [7], unlike a *dosR* mutant [15]. Since DevRS/DosT comprise the DevR signaling pathway and wild-type levels of *dosT* transcripts were detected in Mut1 and Comp1 bacilli (data not shown), a paucity of signaling through the kinases is unlikely to occur in Mut1 or Comp1 strains. Therefore we attribute the hypoxia adaptation defect to the disruption of *devR* function alone and not that of the kinases. We have established in the present study that DevR_N_ is the active inhibitor species in the DevR_N_-Kan fusion protein. Moreover, the kanamycin resistance cassette is routinely used in genetic analysis and is not known to confer any abnormal phenotype to *M. tb*. Therefore, our results establish DevR_N_-Kan as a signaling inhibitor of the DevR-mediated hypoxia response and we exclude an ‘unnatural’ function for the fusion protein in this response. However, the effect of DevR_N_-Kan expression per se on other aspects of bacterial physiology including virulence awaits further investigation.

Importantly, the attenuated phenotype exhibited by Comp1 bacteria was stable and not modulated by animal passaging. Further investigation is necessary to understand the mechanism underlying attenuation of the Comp1 strain. However, the demonstrated repression of WT DevR function by DevR_N_-Kan signaling inhibitor suggests the possibility that such a knockdown approach that intercepts bacterial signaling could be useful for studying and perhaps for modulating the activity and function of other *M. tb* signaling pathways.

## Materials and Methods

The plasmids and strains used in this study are described in Tables 1 and 2, respectively.

### Construction of pAVdevR_N_–Kan

The *devR* gene was disrupted by in-frame insertion of a kanamycin resistance cassette (kan) from pGP1–2 (kind gift from Dr. S. Tabor, USA) at an unique PpuMI site. DevR_N_-Kan fusion protein-coding DNA sequence was cloned downstream of the native operon promoter described earlier [21] to generate pAVdevR_N_ -Kan.

### Preparation of Passaged Bacilli

All experiments were performed with guinea pig-passaged *M. tb* WT, Mut1 and Comp1 strains unless mentioned otherwise. For passaging, −70°C frozen stocks of laboratory cultured bacilli were thawed, resuspended in PBS and ∼5×10^6^ CFU were injected subcutaneously into guinea pigs as described [13]. Bacilli were recovered from guinea pig spleens at 6 weeks post infection by plating on Middlebrook (MB) 7H11 agar with OADC. Bacterial scrapings were cultured in 7H9 medium containing Albumin Dextrose Complex (ADC) and stored frozen at −80°C for further use.

### Bacterial Culture

Frozen passaged bacterial stocks were sub cultured twice or thrice to logarithmic phase (A_595_∼0.4) prior to viability and expression analysis.

### Expression Analysis

Various logarithmic phase *M. tb* cultures were diluted to A_595_ of 0.025 and grown with shaking to an A_595_ of 0.3. A culture aliquot was immediately harvested by centrifugation (aerobic culture). For hypoxic cultures, 10 ml aliquots of aerobic cultures were dispensed into 50 ml screw-capped tubes and kept standing for 5 days. Lysates were prepared as described [27] from two to four independent cultures at each condition. Aliquots containing 10 to 15 µg protein were subjected to immunoblotting using rabbit polyclonal antisera as described [19]. Anti-SigA antibody was a generous gift from Dr. T.S. Balganesh (AstraZeneca, Bangalore). Densitometric analysis was performed using Quantity One software (Biorad, USA). The signal intensities derived from SigA in each lysate were used to normalize the signal intensities from DevR and DevR_N_-Kan.

### GFP Reporter Assay

GFP reporter activity of pOperon-2 and pdevR-2 constructs was assessed under aerobic and hypoxic conditions as described earlier [28]. Briefly, the *M. tb* strains were subcultured twice to mid-logarithmic phase and then 3.3 ml aliquots (A_595_ = 0.1) were dispensed in 5 ml Vacutainer tubes (BD) which were kept standing (hypoxic conditions). GFP fluorescence was measured at the specified time points.

### *In Vitro* Phosphotransfer Competition Assays

Full length DevS (DevS_578_) was purified as described earlier [29]. Full-length DevR was overexpressed and purified from *E. coli* C43 harbouring pAVDevR by standard techniques. DevR_N_ -Kan fusion protein was purified from *E. coli* carrying pKKNKan by standard techniques. DevS_578_ (5 µM) was autophosphorylated using 5 µCi γ - ^32^P-ATP (approximately 3800–4000 Ci/mmole, BRIT, Mumbai, India) in a 20 µl reaction containing 50 mM Tris, pH 8.0, 50 mM KCl, 25 mM MgCl_2_ and 50 µM ATP at 25°C for 60 min as described [7]. DevS_578_∼P was added to a mixture of full-length DevR (5 µM) and DevR_N_-Kan (at concentrations ranging from 0.83 to 30 µM to attain molar ratios of 1∶6 to 6∶1 for DevR∶DevR_N_-Kan, respectively) and incubated for 2 min at room temperature. Samples were electrophoresed through a 15% SDS-PAGE and the gel was subjected to phosphorimaging.

### Assessment of Viability of *M. tuberculosis* Strains *In Vitro*

*M. tb* cultures were diluted to A_595_ of 0.005 and 10 ml aliquots were dispensed in 50 ml tubes and grown either with shaking at 220 rpm (aerobic setup) or kept standing in 15 ml tubes (hypoxic setup). Cultures were sampled once only from separate tubes dedicated for each time point of the hypoxia set up. Bacterial CFU at defined time points was estimated by plating serial dilutions in duplicate on MB 7H11 agar containing ADC and incubating the plates at 37°C for 6 weeks.

### Guinea Pig Virulence Assay

Approval was taken from the Institutional Animal Ethics Committee, NTI, Bangalore prior to guinea pig experiments. Guinea pigs were infected by subcutaneous route with passaged *M. tb* strains in phosphate buffered saline (approx. 5×10^6^ viable organisms per animal) for 6 weeks and 13 weeks (6–10 animals per group) as described [13]. The virulence assay in guinea pigs was performed as described [13], [24], [25]. In this model, bacteria spread to the lungs and spleen from the site of injection (thigh). Briefly, at the time of sacrifice, internal organs were examined for visually scorable lesions in spleen, liver, lung, inoculation site and its draining lymph nodes as described [24]. Lungs and spleens were transferred to selective Kirchner's liquid medium for CFU determination as described [13]. The spleens and right lower lobes of lungs were individually homogenized in dedicated homogenizers and serial dilutions were plated in duplicate on MB 7H11 agar containing OADC and also on LJ slopes. The colonies were counted after 6 weeks of incubation at 37°C. Portions of lung and liver were fixed in 10% formalin and processed for histopathological analysis by staining with haematoxylin and eosin as described previously [30]. Laboratory cultured strains were also assessed in the 6 weeks virulence assay (10 animals per group). The statistical significance of the differences between the various strains for different parameters was determined using the Mann-Whitney test.

### THP-1 Infection Assays

The inocula for infection were prepared by culturing *M. tb* strains with shaking to A_595_∼0.6 in Dubos Tween Albumin. THP-1 cell line was maintained in RPMI 1640 medium supplemented with 10% fetal calf serum and monolayers were prepared and infected as described [31]. Briefly, THP-1 cells were seeded at 0.25×10^6^ cells per well in 24-well tissue culture plates and were differentiated by the addition of phorbol 12-myristate acetate (100 nM) for 24 h. The monolayers were infected with *M. tb* strains at a low m.o.i. (1 bacterium per 50 macrophages) for 20 h, washed with incomplete RPMI 1640. Fresh complete RPMI 1640 was added to each well and the plates were incubated at 37°C for upto 7 days. Intracellular viable bacteria on day 1, 4 and 7 postinfection were assessed by lysis of the monolayers in 0.025% SDS, followed by plating serial dilutions as described above. Infectivity is expressed as a fraction of the number of bacteria internalized on day 1 to the total number of bacilli added. Significance was determined by one-way ANOVA followed by post-hoc analysis using Bonferroni correction.

## Supporting Information

Table S1 (0.05 MB DOC)

Figure S1 Bacterial recovery (Mean plus/minus SD) from guinea pigs infected for 6 weeks with passaged or laboratory cultured M. tb strains. *, * *, P<0.05 in comparison to WT and Mut1, respectively. #, P<0.05 between the passaged and the laboratory cultured strains.
(0.18 MB TIF)
