# Supplementary material for: Co-Expression of DevR and DevRN-Aph Proteins Is Associated with Hypoxic Adaptation Defect and Virulence Attenuation of Mycobacterium tuberculosis
Source: PLoS One. 2010 Feb 26;5(2):e9448. doi: 10.1371/journal.pone.0009448 (PMC2829086; doi:10.1371/journal.pone.0009448)
Supplement: Table S1 — (0.05 MB DOC) [file pone.0009448.s001.doc]

**Supplementary Table S1. Virulence comparison of various *M. tuberculosis* strains**

|  | 6 weeks (Passaged strains) | | |  | 6 weeks (Laboratory cultured strains) | | |
| --- | --- | --- | --- | --- | --- | --- | --- |
|  | WT | Mut1 | Comp1 |  | WT | Mut1 | Comp1 |
|  |  |  |  |  |  |  |  |
| **Visual scores^#^** | 32.25 ± 4.34 | 25.25 ± 5.85 | 8 ± 0*,** |  | 26.1 ± 7.50 | 14.9 ± 6.11* | 11.2 ± 5.18* |
|  |  |  |  |  |  |  |  |
| Lung granuloma (%) | 58.75 ± 2.39 | 43 ± 15.77 | 25 ± 2.04* |  | 44.5 ± 7.47 | 30.5 ± 7.65 | 13.5 ± 3.16* |
|  |  |  |  |  |  |  |  |
| Liver granuloma (%) | 46.25 ± 9.43 | 18.75 ± 3.75 | 12.5 ± 4.78* |  | 23 ± 4.22 | 8.4 ± 1.67* | 5.8 ± 1.46* |
|  |  |  |  |  |  |  |  |
| Spleen weight ratios^ | 1.08 ± 0.25 | 0.87 ± 0.40 | 0.26 ± 0.05*,** |  | 0.58 ± 0.24 | 0.35 ± 0.08* | 0.26 ± 0.08*,** |
|  |  |  |  |  |  |  |  |
| Lung weight ratios | 0.80 ± 0.14 | 0.79 ± 0.05 | 0.73 ± 0.14 |  | 1.14 ± 0.38 | 0.88 ± 0.14 | 0.95 ± 0.24 |
|  |  |  |  |  |  |  |  |
| Liver weight ratios | 5.85 ± 0.37 | 4.51 ± 0.66 | 5.48 ± 0.92 |  | 3.91 ± 0.58 | 3.87 ± 0.64 | 3.72 ± 0.60 |

^#^ Mean total of scores assigned to spleen, liver, lung and the site of injection along with its draining lymph nodes immediately after death as described (24).

* represent P<0.05 in comparison to WT.

** represent P<0.05 in comparison to mut1.

^ Weight ratio = (organ weight/ body weight) × 100.
