# Supplementary figures and images for: Co-Expression of DevR and DevRN-Aph Proteins Is Associated with Hypoxic Adaptation Defect and Virulence Attenuation of Mycobacterium tuberculosis
Source: PLoS One. 2010 Feb 26;5(2):e9448. doi: 10.1371/journal.pone.0009448 (PMC2829086; doi:10.1371/journal.pone.0009448)

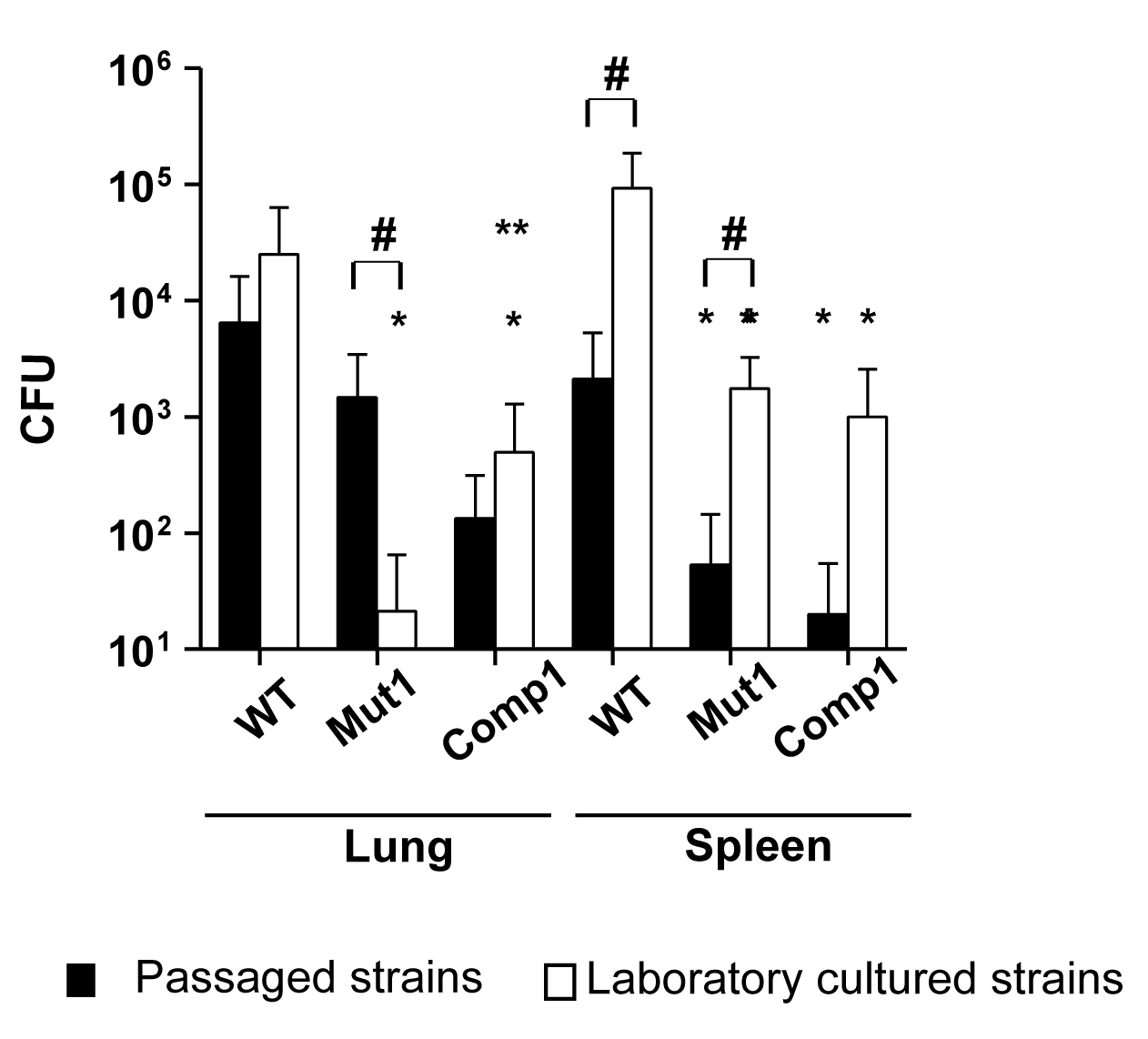

Supplement: Figure S1 — Bacterial recovery (Mean plus/minus SD) from guinea pigs infected for 6 weeks with passaged or laboratory cultured M. tb strains. *, * *, P<0.05 in comparison to WT and Mut1, respectively. #, P<0.05 between the passaged and the laboratory cultured strains. (0.18 MB TIF) [file pone.0009448.s002.tif]
